# A qualitative study on the experience of internet outpatient consultation in palliative care for relatives of cancer patients

**DOI:** 10.1186/s12904-025-01732-3

**Published:** 2025-04-04

**Authors:** Shuo Li, Guijun Lu, Yongqing Shen, Jianya Ye, Jingmin Ji

**Affiliations:** 1 School of Nursing, Hebei University of Chinese Medicine, Shijiazhuang, 050200 Hebei China; 2 Department of nursing, Beijing Tsinghua Changgung Hospital, School of Clinical Medicine, Tsinghua Medicine, Tsinghua University, Beijing, 102218 China; 3 Key Laboratory for Health Care with Chinese Medicine of Hebei Province, Shijiazhuang, 050200 China

**Keywords:** Relatives of patients with malignant tumors, Home-based palliative care, Experience, Internet-based outpatient clinic, Qualitative study

## Abstract

**Background:**

The palliative Internet-based outpatient service ensures seamless connectivity and equitable patient care; however, there is a lack of research on service quality, as well as the experiences and perceptions of patients’ relatives. The aim of this study was to explore the perspectives and experiences of patients’ relatives when using the palliative Internet clinic.

**Methods:**

A qualitative study involving semi-structured interviews with 13 relatives of terminal cancer patients was conducted, and data were analyzed using content analysis.

**Results:**

Relatives reported that they were capable of caring for the patients at home but experienced confusion and challenges in managing their symptoms and addressing their psychological issues. They expressed hope that Internet clinics would provide timely and effective professional guidance to families, easing caregiving burdens and enhancing the quality of home-based care.

**Conclusions:**

This study highlights the potential of Internet-based palliative care to enhance the quality of home-based caregiving and reduce caregiver burden. In addition, Internet-based consultations as an alternative to face-to-face consultations offers an appropriate option in palliative care settings.

## Background

Tumors are a major global cause of death, placing significant strain on medical resources worldwide [1]. Patients with advanced-stage tumors often incur high treatment costs and experience significant suffering before passing away [2]. Early palliative care for individuals with terminal cancer provides significant benefits for both patients and their caregivers [3]. An increasing number of end-stage cancer patients prefer home palliative care [4, 5] However, there are challenges in providing effective and comprehensive care services [6]. The emergence of Internet-based palliative care offers potential solutions to these challenges [7]. With the rapid advancement of technology, innovative technologies have permeated palliative care. Patients and families can confer online via Internet-based palliative care to reduce the number of hospital visits, save healthcare resources, prevent in-hospital fatalities [8, 9], reduce the symptom burden, and improve the quality of life of cancer patients [10, 11]. Internet-based palliative care is crucial for rural areas and patients with limited mobility. It enhances accessibility by delivering remote consultations and follow-ups, decreasing the need for lengthy travel durations [12]. However, most home palliative patients are critically ill or elderly, and they have difficulty using cell phones or the Internet to contact healthcare providers [13]. Family support is especially important, as healthcare providers need to assess the condition of patients with declining physical abilities and cognitive language impairments through their family members. Therefore, when implementing remote home palliative, patients need to be accompanied by a caregiver who understands the patient’s condition and knows how to provide care [14]. This often causes significant stress for their relatives, who were eager to effectively communicate with their healthcare providers and receive help in managing the disease symptoms [15]. It is important for medical staff to fully understand the perspectives of family members participating in Internet-based palliative care to deliver targeted services. Understanding these factors enables healthcare providers to deliver the necessary care that addresses the needs of both patients and their family members. This study aims to explore the experiences and perspectives of patients’ relatives when visiting the palliative Internet clinic, as well as the demand for counseling services offered by Internet-based outpatient clinics among patients’ relatives.

## Methods

### Philosophical foundations and research methodology

We used the descriptive-natured qualitative research methodology, which is philosophically grounded in naturalistic questioning and whose principle is to recognize the diversity and interactive, inextricable nature of the common experience of human communication [16]. The aim is to provide a rich, direct description of an event. We choose descriptive research methodology to examine the experiences and views of patients’ relatives when visiting the palliative Internet clinic.

### Participants and recruitment

In this study, the relatives of cancer patients who registered online in the palliative outpatient clinic of our hospital from February to April 2023 were selected as the research objects by a purposive sampling method.

Patients’ relatives were included in this study if they attended the palliative Internet clinic, were willing to use Internet video call software, were immediate family members, were familiar with the patient’s condition, and personally cared for the patient. Participation in the study was voluntary. We excluded individuals who were not relatives of patients and who were not first-time patients. The sample size was determined based on reaching data saturation, where no new information emerged. Thirteen interview subjects were finally included in this study. For relatives, we will use numbers (A to M) instead of their real names. The study was approved by the Ethics Committee of Beijing Tsinghua Changgeng Hospital (reference 23331-0-01). All study participants gave written informed consent for participation, data processing, and publishing.

### Data collection

We reviewed the existing literature, then found that the primary concern in the previous literature was determining the types of services from an online clinic [17], which aligns with the goal of our research. We developed a preliminary interview outline, two participants pre-tested the preliminary interview version to ensure the questions were relevant and understandable. Based on the results of this pre-interview, we consulted with two palliative care specialists and revised the outline to develop semistructured interview questions (Table 1). Consent was obtained from the relatives of patients to conduct one-on-one, semi-structured, in-depth interviews after their Internet consultations using the Jingyitong Internet-based Medical Software. Before the interviews, the researcher explained the study’s purpose, content, and other relevant information using the interview outline to the patient’s family. During the interviews, techniques such as questioning, follow-ups, responding, and active listening were employed, along with restating, clarifying, and summarizing, to ensure the accuracy of the information gathered. The entire process was recorded.

**Table 1:** Semistructured interview questions

In	Interview questions
1	What specific areas of concern would you like to address through the palliative Internet clinic? Can the Internet consultation meet your needs and alleviate your concerns?
2	What are your expectations of the palliative Internet clinic?
3	What assistance would you most like to receive from the Internet clinic?
4	Would you recommend the palliative Internet clinic to others? If so, what are your reasons for doing so?
5	What other suggestions or additions do you have for the palliative Internet clinic?

### Data analysis

One researcher transcribed the audio recordings verbatim into text within 24 h after each interview. The text was transcribed and reviewed, and any unclear sections were referred to the patient’s family for clarification. The researcher eliminated personal biases and previous experiences during data analysis, focusing solely on the interviewers’ responses. The team members worked together, and specialists communicated and supervised to ensure the study’s credibility and reliability [18].

We utilized Nvivo 11.0 analysis software for transcribing and importing the data and employed the targeted content analysis method for data analysis [19, 20]. (a) We extracted relevant essential concepts and variables as initial coding categories based on the previous literature, then provided operational definitions for each category; (b) Read the interview data verbatim, marked those that are closely related to the research questions; (c) Coded and categorized the content using the initial coding scheme, assigned new codes to encodable content, next made adjustments and modifications to the existing coding scheme; (d)

Codes were categorized into taxa and sub-taxa based on the degree of relevance and association of the codes; (e) Organize and conceptualize categories to form themes. Researchers should engage in continuous comparison and discussion during data analysis to ensure consistency in the content of the data collected.

## Results

A total of 13 patient family members were interviewed (Table 2), each lasting between 45 and 116 min, resulting in an overall interview duration of 985 min.The following two themes and 14 sub-themes emerged from the compilation and analysis of the interview data(Table 3).

**Table 2:** General information of respondents and patients(*n* = 13)

number	kindred							patient		
	Sex	Age range(years)	Relationship to patient	Educational level	residence	Have you heard of palliative before counseling		Sex	Age range(years)	diseases
A	female	60–65	mother-daughter	high school	Beijing	yes		female	90–95	breast cancer
B	female	45–50	father-daughter	high school	Jiling	no		male	75–80	intrahepatic cholangiocarcinoma
C	male	70–75	father-son	undergraduate	Changchun	yes		male	40–45	colorectal cancer
D	male	55–60	mother-son	undergraduate	Zhejiang	yes		female	75–80	liver cancer
E	female	50–55	mother-daughter	specialized subject	Beijing	yes		female	75–80	lung cancer
F	female	40–45	(older and younger) sisters	specialized subject	Beijing	yes		female	45–50	breast cancer
G	female	35–40	mother-daughter	high school	Beijing	yes		female	60–65	lung cancer
H	female	60–65	father-daughter	undergraduate	Beijing	no		male	80–85	lung cancer
I	male	40–45	father-daughter	master’s degree	Beijing	yes		male	65–70	colorectal cancer
J	female	55–60	father-daughter	specialized subject	Beijing	no		male	80–85	liver cancer
K	female	45–50	brother and sister	undergraduate	Beijing	yes		male	50–55	liver cancer
L	female	55–60	(older and younger) sisters	undergraduate	Dalian	no		female	60–65	gastric cancer
M	female	55–60	brother and sister	undergraduate	Hebei	no		male	60–65	liver cancer

**Table 3:** Themes and subthemes

Themes	Subthemes
Difficulties and dilemmas in-home care for relatives of patients with malignant tumors	1.Pain
	2. Abdominal bloating
	3.Dyspnea
	4.Fatigue
	5.Plumbing care
	6.Wound care
	7.Dietary requirements
	8.End-of-life symptom recognition
	9.Emotional problems
Needs and concerns of relatives of patients with malignant neoplasms on their patients’ hospitalization in palliative	10. Palliative unit admission and discharge condition consultation
	11.Waiting time for admission to a palliative unit
	12.Hospitalization environment requirements
	13.Methods of patient transportation
	14.Need for religious beliefs

### Difficulties and dilemmas in-home care for relatives of patients with malignant tumors

#### Pain

Pain is a prevalent symptom of terminal cancers. When not managed effectively, it can significantly reduce the patient’s quality of life and create distress for their relatives.

“*My sister has been on pain medication*,* but she still wake up in pain at night. Is it possible to ease her evening pain so she could sleep better?“(F).*

*“My dad is unable to eat right now because of the pain. Is there another method to alleviate the pain*,* what pain relief options are available for patients in palliative care?“(I).*

#### Abdominal bloating

Abdominal distension is a clinical symptom often caused by tumor growth and is particularly common in patients with advanced tumors. This condition frequently hampers the patient’s breathing, as noted by their relatives.

*“My father hasn’t had a bowel movement in several days and often complains of bloating. What is the best way to help him have a bowel movement?“(J).*

*“Brother’s belly is so large that he can’t lie flat and struggles to breathe*,* appearing very uncomfortable.“( K).*

*“It seems like my brother has a hard lump in his stomach that occasionally hurts*,* and we don’t know what to do about it.“(M).*

#### Dyspnea

Dyspnea is the most common symptom in end-of-life patients, typically leading to increased agitation and psychological stress for caregivers.

*“The older man also uses oxygen at home but feels it does not alleviate his choking.” (H).*

*“My mother currently has a chest tube that needs daily drainage*,* but she continues to struggle with her breathing.” (G).*

*“We tried several approaches*,* but none of them corrected his choking problem. We are concerned that he may not survive.” (L).*

#### Fatigue

Fatigue reflects the depletion of skeletal and visceral muscle mass, which cannot be fully restored through nutritional support. The patient’s relatives describe the patient as indifferent and uninterested in participating in activities.

*“I feel like my brother sleeps all day and doesn’t say a word.” (M).*

*“My mom used to be able to stand up and eat*,* but in the last few days*,* she has just been lying in bed and barely eating.” (D).*

*“My mother often feels weak; even after eating*,* she stays in bed all day*,* and her mood is low.“(E).*

### Plumbing care

Various drainage devices, such as central venous catheters, urine catheters, stomach tubes, chest drains, and abdominal drains, often restrict a patient’s physical activities. Additionally, the lack of nursing knowledge and skills related to these drains among family members can increase the stress of caregiving.

*“The patient has a chest tube that is drained daily*,* but the patient still feels breathless.“(G).*

*“My father has a bile duct drainage tube. He has trouble sleeping every night because he is frightened of touching it. Can the tube be withdrawn without affecting his life?“(B).*

*“I don’t know what to do because my mother’s tubes are getting bigger and bigger. I’m really anxious about taking care of her.“(D).*

### Wound care

Persistent wounds, such as malignant wounds and pressure injuries, pose significant risks to a patient’s physical health. They also create emotional distress for both the patient and their family. Additional symptoms, including pain, odor, bleeding, and leaking, can intensify the psychological burden on patients and their loved ones, making them feel overwhelmed as they manage and care for these wounds.

*“My mom has a bedsore on her hip*,* and there is always some fluid coming out. What should I do to help her with it?“(E).*

*“My sister’s surgical wound developed another pustule that had ruptured*,* causing it to hemorrhage and exude fluid with the slightest contact. It often generated a horrible stink*,* but she always refused whenever I tried to help my sister clean it.” (F).*

*“My brother’s wound has not healed since his surgery. Initially*,* we didn’t take it seriously*,* but now the cut is worsening*,* and there are many small bumps appearing around it. Could you please advise me on how to help him deal with this?” (K).*

### Dietary requirements

Providing dietary guidance is crucial in implementing palliative care. Effective dietary advice can help families manage the patient’s food intake, enhance the patient’s nutritional status, and alleviate the psychological burden on relatives.

*“My child used to adore their mother’s dumplings*,* but he now has gastrointestinal troubles. Is it still safe for him to consume those dumplings?” (C).*

*“Now that my sister has a stomach tube*,* she misses the taste of coffee.” Is it okay for her to consume coffee?“(L).*

*“My brother has not had a bowel movement in days and refuses to eat. Is there any way to get him to eat something?“(M).*

### End-of-life symptom recognition

Non-medical relatives may find it challenging to accurately identify when a patient is in the terminal phase of life due to the individual variations in how patients present at this stage.

*“What are the signs that indicate a patient is dying?“(K).*

*“I wonder if the patient’s lethargy is a sign that he is going to die.“(M).*

*The patient cannot speak*,* yet surprisingly*,* he spoke yesterday. Could it be a flashback?“(L).*

### Emotional problems

As patients approach the end of life, they often experience a range of emotions, such as frustration, panic, worry, and sadness. Inner psychological struggles, including feelings of “unwillingness,” “inability to let go,” and “reluctance,” can lead to emotional distress for both the patients and their families. This turmoil can result in deep grief that affects the entire family unit.

*“I don’t know how to face the days ahead.“(A).*

*“My parents are unaware of my brother’s health*,* and I’m uncertain about how they’ll react when they find out. I’m not sure how to express what’s going on right now.” (K).*

*“Since Mom and Dad had limited opportunities to see each other*,* I often witnessed Mom crying alone at home.“(J).*

### Needs and concerns of relatives of patients with malignant neoplasms on their patients’ hospitalization in palliative

#### Palliative unit admission and discharge condition consultation

Palliative care units in China do not have clearly defined criteria for patient admissions. For patients with non-malignant tumors, admission criteria are based on factors such as disease duration, symptoms, and laboratory indicators. However, there are no specific criteria for patients with malignant tumors. Family members often express skepticism and concern regarding the patient’s admission to palliative care and the quality of long-term care provided.

*“Other hospitals refused to take us*,* claiming that we no longer needed therapy. Can you admit patients?“(A).*

*“My brother was in a palliative unit at the hospital*,* but he was only allowed to stay there for two weeks.” (K).*

*“I do not know how much longer my brother will live*,* and if his condition continues to worsen*,* will he be able to remain in your palliative unit?” (M).*

*“Families may be uncertain about the circumstances that warrant palliative admission and whether a patient can still be discharged from the hospital after being admitted to palliative.“(H).*

### Waiting time for admission to a palliative unit

Many medical institutions in China have established palliative care wards; however, hospitals often experience a shortage of palliative care beds. These wards prioritize a family-oriented and individualized approach, distinguishing them from standard wards. As a result, many patients in need of palliative care find themselves waiting for a bed. Relatives often feel anxious about the uncertainty of wait times, fearing that their loved ones may suffer or miss out on essential palliative treatment.

*“My father died from lung cancer not long ago. At the time*,* I went to numerous hospitals to arrange for a bed*,* but they all said we had to wait. He ultimately died in a nursing home because we were unable to acquire a bed in time. Now I’m anxious about whether my sister will be able to acquire a palliative bed when she needs one.“(F).*

*“I haven’t looked into local hospitals that accept patients with terminal tumors*,* let alone hospice wards. Some acquaintances led us to various hospitals in Beijing that can accept such patients*,* and we visited each one for consultation. However*,* they advised us that we would have to wait for a bed and were unable to specify a specific wait time. Is there a way to reserve a bed? We’d like to make a reservation in advance so that when a bed becomes available*,* we can be there as quickly as possible.“(C).*

### Hospitalization environment requirements

The palliative unit has both single and shared rooms. Regardless of the type of room, the ward environment should make patients feel at home, satisfy their senses, safeguard their privacy and dignity, and support their social interactions and spiritual expressions. Some patients preferred a private room for privacy, quiet, and better sleep. They were concerned about disturbing other patients and feeling embarrassed for difficult symptoms. These challenges can include difficulties with sleeping and eating, as well as the need for specialized care for stomas or wounds. All of which create unique demands on the hospital environment.

*“We used to live in a shared room*,* but the noise from other patients at night disturbs my mother’s sleep. I was wondering if there were any private rooms available at your hospice?” (A).*

*“Because my father enjoys peace and quiet*,* he would prefer a private room.“(B).*

*“Because my mother frequently needs to use the bathroom at night*,* I hope that patients can have single rooms to avoid disturbing others.” (G).*

*“My father has an intestinal stoma in his stomach*,* and there is an odor when he changes the stoma. I’ve had horrible encounters with other patients due to the stench of changing the stoma*,* so I’d like him to have a private room*,* ideally a private one.“(I).*

### Methods of patient transportation

Relatives encounter several obstacles and concerns when it comes to transporting terminally sick patients. Common concerns include limited patient mobility and limited access to emergency supplies, especially when emergency services are hesitant to transfer end-of-life individuals.

*“My father has tubes attached to his body*,* and I don’t know how to get him to the hospital.“(J).*

*“I’m not sure how my disabled father can get up the stairs without an elevator. Can you offer transportation for him?” (I).*

### Need for religious beliefs

Religious beliefs are a distinct social ideology and cultural phenomenon, providing spiritual solace to patients and their families. Religious beliefs can provide vital spiritual support to patients reaching the end of their lives, allowing them to find peace and tranquility at this challenging time.

*“My father was a devout Buddhist.” “Can we perform a puja in the hospital room to send him off after his death?” (I).*

*“Does the hospice offer any religious ceremonies for patients? Can they provide a venue?“(B).*

## Discussion

China’s online nursing clinic has grown, including intravenous catheter maintenance, wound treatment, diabetes control [21]. Currently, Internet palliative clinics are in the experimental phase [22]. This study utilized qualitative methods to gain insights into the experiences and emotions of relatives of cancer patients, which are crucial for developing Internet palliative clinic services. The findings reveal that oncology patients require guidance and support from Internet palliative clinics for their home care, aligning with the results of other studies [23, 24]. Internet palliative clinics place greater demands on healthcare providers’ professional competence and overall quality than traditional clinics [25]. Relatives expect these providers to deliver proper treatment for physical symptoms, comfort care, and psychosocial support, as well as to offer the option of hospitalization in a palliative unit. This indicates that family members feel pressured to provide care, even though most patients would prefer to spend their final days at home [26, 27, 28]. Families are increasingly prioritizing care counseling to support patients in their daily lives, sush as managing pain and diet [25, 29]. This guidance can be provided online to help relatives care for patients. Additionally, in addition to pain and diet management counseling, we discovered that other critical parts of care require attention, such as drainage management, breathing difficulties, and other specialized, individualized care challenges. Online consultations will become more comprehensive and tailored to offer individualized care guidance for caregivers [30]. Palliative care nurses working with Internet-based outpatient services need to have extensive experience in caring for patients who are nearing the end of life. They should be skilled in making medical decisions related to therapy, prognosis, and assessing the severity of the patient’s illness. Additionally, these nurses need to possess specific social, psychological, and spiritual counseling skills to ensure timely treatment for patients who may have medically treatable conditions [31, 32]. As a result, palliative outpatient nurses need to receive training in oncology and other terminal illnesses. They need to also understand the role of medical social workers and be able to refer patients to social services in a timely manner [33].

This study discovered that the relatives of the patients lacked the knowledge and ability to deliver care. This can be enhanced by utilizing multimedia and video technology to relieve patients’ unpleasant sensations and minimize physical and emotional stress [25]. Patients with advanced tumors frequently encounter various psychological challenges, such as anxiety, feelings of helplessness, and sadness. Similarly, family members frequently experience significant emotional distress as they witness the suffering of their loved ones. They often feel uncertain about the most effective ways to provide emotional and practical support.

The palliative Internet clinic offers quick and straightforward online assistance and support for the relatives of patients. It should provide referral services and expert assessments to help those who are experiencing significant psychological distress and a lack of social support [34].

This home palliative model combines online counseling with in-person visits, providing a novel service strategy for patients while efficiently addressing the limitations of standard palliative treatments [35]. This is identical to the foreign “TapCloud” “ASyMS” remote platform, which is equally effective at managing health to suit the medical demands of palliative home patients. It also reduces the physical and mental stress of the patient’s family members and boosts the confidence of relatives providing care [36, 37].

During the screening process for palliative care, nurses may notice that the patient or their family requires more psychosocial assistance. They might work with a multidisciplinary team to provide multi-dimensional care for home-based palliative care patients [38]. However, establishing Internet consultations presents various problems, particularly for senior patients. They may have difficulty using communication devices and experience network problems in isolated rural places with little coverage. These limitations limit the effectiveness of Internet consultations. Medical staff should encourage family members to participate and assist senior patients in communicating with medical staff online [39].

The increasing usage of online nursing clinics has dramatically increased the accessibility and convenience of nursing care. However, most of these services are now limited to a single hospital, primarily providing pre-hospitalization and post-discharge care for patients [28, 39]. Palliative patients, especially those with terminal illnesses, have unique and demanding needs. These challenges include their unstable health status, mobility issues, and the complexities involved in their care. To better address these needs, we require a comprehensive and integrated service delivery system. This system should implement a standardized continuum of care provided by multidisciplinary teams to ensure that patients receive consistent care across all stages and settings. Additionally, it is essential to establish inter-regional and national referral processes for palliative patients. This will facilitate smooth transfers to the most suitable healthcare facilities based on their specific conditions and requirements. An online platform can serve as a bridge between medical institutions, communities, and families, enabling the sharing of information and the integration of resources. We aim to create a national hospice passport care system that connects hospitals, communities, and home care, thereby improving accessibility and continuity of hospice services in tertiary hospitals.

Given that our study focused on the feelings and needs of relatives caring for cancer patients, it did not address whether relatives were concerned about accessing Internet-based outpatient services. This omission may limit the generalizability of our findings. Future research should develop a quality rating system for Internet-based palliative care in conjunction with quantitative studies. This approach would enhance the development of Internet outpatient services while better meeting the needs of caregivers.

## Conclusion

In this study, we employed a descriptive phenomenological research method to investigate the experiences of relatives of cancer patients who consulted with the palliative Internet clinic. The findings revealed that while family members acknowledged the critical importance of managing patients’ symptoms, they encountered several challenges and psychological burdens during the caregiving process. Specifically, relatives identified a range of care needs, including effective symptom management, psychological support for patients, identification of end-of-life symptoms, and guidance on hospitalization decisions. They believed that an Internet platform would provide them with timely and thorough information, as well as skill support, to help them manage these challenges. In light of these findings, future palliative Internet clinics should consider offering more extensive and personalized care instructions to relatives. This approach would enhance their capacity to provide both effective and compassionate care to patients.

## Data Availability

No datasets were generated or analysed during the current study.
